# Are people willing to take regular vaccinations? A qualitative study among diverse ethnic groups in Hong Kong

**DOI:** 10.1371/journal.pone.0318631

**Published:** 2025-05-02

**Authors:** Yan Li, Ivy Yan Zhao, Wenze Lu, Sau Fong Leung, Daniel Bressington, Lin Yang, Yao Jie Xie, Mengqi Li, Angela Y. M. Leung

**Affiliations:** 1 School of Nursing, the Hong Kong Polytechnic University, Hong Kong, SAR, China; 2 Faculty of Nursing, Chiang Mai University, Chiang Mai, Thailand; National Institutes of Health, University of the Philippines Manila / De La Salle University, PHILIPPINES

## Abstract

Vaccine hesitancy is identified as one of the top ten global health threats by the World Health Organization. The emergence of new coronavirus variants and evidence of waning immunity offered by COVID-19 vaccines draw attention to the need for regular vaccinations. This study aimed to understand the people’s hesitancy towards regular COVID-19 vaccination among diverse ethnic groups of citizens in Hong Kong. A qualitative descriptive research approach was adopted. Content analysis was used for data analysis. A purposive sample of fifteen participants, aged 20–75, with diverse ethnicities (Chinese, Palestinian, British, French and Indian) joined the study. Their barriers and hesitancy toward taking the COVID-19 vaccination included personal beliefs and biases, policies and ethical considerations, and concerns about vaccines and vaccination. Collaborative efforts of governments, health policymakers, healthcare providers, media sources and vaccine manufacturers are required to decrease vaccine hesitancy. Cultural values and ethical issues should be considered in vaccine promotion. This study contributes to the existing body of literature by exploring vaccine hesitancy among ethnically diverse populations during the COVID-19 pandemic. The findings provide critical insights into the underlying reasons driving vaccine hesitancy among ethnic groups in Hong Kong and support the development of targeted public health strategies to address this issue. Public health researchers and practitioners are encouraged to prioritize addressing mistrust and ethical concerns while leveraging the strengths of multicultural communities and fostering coordinated efforts among diverse organizations to reduce vaccine hesitancy against future global health challenges.

## Introduction

Vaccine hesitancy has been recognized by World Health Organization [1] as one of the top ten global health threats. It generally refers to ‘*delay in acceptance or refusal of vaccines, despite the availability of vaccine service*’ [2]. It is increasingly understood as a complex and dynamic process that reflects uncertainty or indecision about vaccination, rather than a fixed position of acceptance or refusal [3]. Some studies have indicated that vaccine hesitancy is distinct from the vaccination behavior of rejection, as individuals who ultimately decide to vaccinate can still experience hesitancy [3, 4]. They have considered vaccine hesitancy as a state of indecision or a psychological state that exists independently of the final vaccination decision.

The emergence of new coronavirus variants and evidence of waning immunity conferred by COVID-19 vaccines highlight the need for regular vaccinations to sustain population immunity. However, achieving widespread vaccine uptake remains a challenge, as hesitancy continues to influence vaccination decisions globally [5]. According to the Vaccine Hesitancy Determinants Matrix Model, vaccine hesitancy is influenced by a complex interplay of contextual factors (e.g., historical, cultural, environmental, institutional, economic, and political), individual and group influences (e.g., personal beliefs and social dynamics), and vaccine-specific considerations (e.g., awareness, safety, and efficacy doubts) [2,6].

Global studies have provided valuable insights into the factors driving COVID-19 vaccine hesitancy. For example, Troiano and Nardi [7] conducted a review of vaccine hesitancy studies during the COVID-19 pandemic and identified key reasons for hesitancy, such as general distrust of vaccines, concerns about the safety of COVID-19 vaccines, perceptions of the disease as harmless, and doubts about vaccine efficacy. Similarly, Begum et al. [5] synthesized studies on COVID-19 vaccine hesitancy across diverse cultural and social contexts, finding that hesitancy was particularly evident among certain demographic groups, including young people, females, non-medical students, and healthcare workers. Regional studies further highlight the localized nature of vaccine hesitancy. For instance, in Turkey, the hesitancy was attributed to insufficient knowledge about vaccines and mistrust in their ability to prevent disease [8]. In Malaysia, significant factors included safety concerns, reliance on traditional medicine, and a lack of transparent data [9]. In Taiwan, Confucian collectivism and political partisanship aligned with government policies were found to influence supportive attitudes toward vaccination [10].

Even after the COVID-19 pandemic, such vaccine hesitancy persists and has extended to influence attitudes toward other vaccination programs. Alghalyin et al. [11] reported that worries and misinformation about COVID-19 vaccines contributed to hesitancy toward future influenza vaccination in Saudi Arabia. In the Chinese mainland, Cao et al. [12] found that parental hesitancy toward seasonal influenza vaccination for children was influenced by factors such as vaccine safety concerns and the pervasive role of misinformation on social media, similar to what was experienced during the COVID-19 pandemic [12]. The persistence of COVID-19 vaccine hesitancy has also affected how governmental bodies respond to declining vaccination rates. In Canada, for example, lower vaccination uptake during the COVID-19 pandemic prompted the government to increase the accessibility of pharmacies for administering seasonal influenza vaccines after the pandemic [13]. Policies were introduced to expand vaccination services, promote diverse influenza vaccination programs, and address logistical barriers to improving vaccine uptake [13]. The ongoing influence of COVID-19 vaccine hesitancy on both public attitudes and institutional responses highlights its long-term impact on vaccination behaviors in the post-pandemic era.

However, most existing studies on vaccine hesitancy focus on general populations, often overlooking how cultural values, social norms, and trust in health systems vary across different ethnic groups. The intersection of ethnic diversity and vaccine hesitancy remains underexplored, particularly in multicultural urban centers like Hong Kong, where Eastern and Western influences converge. Its ethnically diverse population includes both local Chinese residents and expatriate communities, each with distinct perspectives on vaccination shaped by their cultural backgrounds and experiences. The population of ethnic minorities in Hong Kong increased significantly by 37% over the past decade [14], and some minority groups may encounter barriers to vaccination. For instance, Southeast Asian minorities may encounter barriers such as language differences, socioeconomic inequalities, and limited access to culturally sensitive health information, which may further exacerbating vaccine hesitancy [15]. Understanding how these diverse populations perceive and respond to regular COVID-19 vaccinations is essential for developing equitable and effective public health strategies.

COVID-19 vaccines were firstly available in Hong Kong since 2021. During the 5^th^ wave of COVID-19 from December 31 2021 to April 3 2022, the statistics from Hong Kong government show that over 85% of the Hong Kong population has received the 2nd dose, and nearly 40% received the 3^rd^ dose [16]. Vaccination uptake is notably lower among individuals aged 70 and above, with fewer than 40% receiving a third dose, and among children under 11 years, with only about 20% completing their second dose [6,17]. Between January and March 2022 in Hong Kong, most COVID-19 deaths occurred among individuals aged ≥60 years, 70% of whom were unvaccinated [18]. The risk of death in this age group was 21.3 times higher for unvaccinated individuals compared to those with 2–3 vaccine doses [18]. The emergence of new variants and evidence of waning immunity highlight the need for ongoing vaccination efforts, particularly among vulnerable populations. Meanwhile, various strategies have been adopted to address vaccine hesitancy, such as the development of communication or information tools for parents/ healthcare workers, advocacy campaigns, and reminder-recall systems [2]. However, previous strategies addressing vaccine hesitancy focused on tackling issues such as knowledge deficits, misinformation, safety concerns, distrust, and religious or philosophical objections through educational approaches and other strategies [2]. Research has demonstrated that solely educating individuals about vaccine safety and efficacy is inadequate to effectively mitigate vaccine hesitancy [6,19].

To restrict the spread of the COVID-19 virus and ensure regular vaccinations, it is important to understand and investigate people’s genuine concerns and worries about vaccine hesitancy in the context of Hong Kong, particularly among diverse ethnic groups [17]. In-depth individual interviews are necessary to explore the specific concerns individuals have regarding COVID-19 vaccines [19]. Therefore, this study aimed to explore vaccine hesitancy among ethnically diverse groups in Hong Kong, with a particular focus on their concerns, motivations, and barriers to regular COVID-19 vaccination. The study sought to advance understanding and address vaccine hesitancy in ethnically diverse populations, while contributing to the development of robust, culturally sensitive, and context-specific strategies to mitigate future public health crises.

## Materials and methods

This is a qualitative descriptive study that aims to capture and interpret participants’ perspectives, which were obtained through semi-structured individual interviews conducted either via telephone or face-to-face, contingent upon adjustments to social distancing measures. We utilized the Consolidated Criteria for Reporting Qualitative Research (COREQ) checklist [20] to report the study.

A purposive sampling method was used to ensure a maximum variation sampling in terms of participants’ backgrounds, such as age, gender, ethnicity, work experience, position, and education level to obtain specific data for our analysis [21]. Adults (at least 18 years) who spoke Chinese or English and were unwilling to take a booster dose of COVID-19 vaccines or future regular vaccines were eligible for inclusion. A bilingual recruitment poster (Chinese and English) was disseminated through media to recruit eligible participants. The recruitment phase was conducted between March and May 2022. Those interested in the study were directed to make contact with the first author. All eligible participants agreed to participate in the study.

Ethical approval was obtained from the Hong Kong Polytechnic University Institutional Review Board (IRB) (Reference Number: HSEARS20210813003). An information sheet was provided to participants and informed written consent from each participant was obtained before commencing data collection. Participant numbers were assigned and used to ensure the anonymity of the participants when reporting the findings. Paper and digital documents of participants, such as audiotapes, transcripts, and notes were kept in a secure and encrypted dresser, with access only given to authorized persons [22].

A semi-structured interview guide (See Appendix 1) was developed based on the Vaccine Hesitancy Matrix model [23] and the relevant literature [2]. An expert panel of four nurse academics and four nurse specialists reviewed the interview guide and comments were addressed accordingly. Pilot interviews were conducted with three Hong Kong citizens before undertaking data collection. Both approaches were designed to ensure we captured a rich and in‐depth understanding of the phenomenon of interest. All the interviews were conducted for 30–45 minutes in either English or Cantonese by a female trained research assistant and digitally recorded. The research assistant received training from the researchers of this study with good qualitative interview experience. Interviews with English speaking participants were independently transcribed verbatim in English by the authors and a trained research assistant. The interviews with Cantonese speaking participants were transcribed verbatim in Chinese by the trained research assistant. Any inconsistency was independently checked by the second author and resolved through discussion with the study team. Transcripts were returned to participants to seek verification of the content. Changes were made to ensure the accuracy of source data.

The content analysis used in the qualitative data analysis followed Elo and Kyngäs (2008)’s three steps framework [24]. The Vaccine Hesitancy Determinants Matrix served as a guiding framework throughout the analysis, structuring the grouping of codes and interpretation of data [23]. All interview transcripts were carefully reviewed against the original audio recordings by the authors to confirm that the transcripts precisely captured the participants’ responses, including subtle nuances in language and meaning. The research assistant who had conducted the interviews was responsible for the verification, leveraging her familiarity with the context of the conversations to maintain the integrity of the data. To enhance the reliability of coding, two authors conducted independent analyses of the data. Each author performed a detailed, line-by-line reading of the transcripts to identify and highlight emerging themes. After independently deriving themes, the two authors refined their interpretations by comparing their findings and identifying areas of overlap and divergence. Regular discussions among all authors were conducted to resolve discrepancies, critically evaluate the coding framework, and reach consensus on the final themes and sub-themes. Member-checking was also incorporated by sharing preliminary findings with a subset of participants to confirm that the themes and interpretations accurately reflected their perspectives. Feedback from participants was integrated into the iterative process of refining the thematic framework.

Information power was utilized in this study to assess the richness of relevant information obtained from the samples [25]. The research team engaged in regular discussions and reviewed interview codes and themes until no new topics emerged, indicating that the optimal sample size had been reached and data saturation achieved [26]. The team concluded that thematic saturation was reached after conducting 15 interviews. Following the 15th interview, it became evident that additional data did not yield new insights, as the identified themes were consistently recurring and well-developed.

## Results

### Summary of demographic data

Of the 15 participants who completed a semi-structured in-depth interview, 11 were female and 4 were male. Table 1 shows their demographic information. They represented different age groups ranging from 20 to 75 years old. Most of them (n = 11) are Chinese and four participants are Palestinian, British, French and Indian. Most of them (n = 9) believed it was a duty/responsibility for them to follow the government’s advice to take the COVID-19 vaccine.

**Table 1:** Demographic summary of interview participants (n = 15).

Characteristics		n
**Age**	18–29 years	5
	30–39 years	4
	50–59 years	4
	60 or above	2
**Gender**	Female	11
	Male	4
**Nationality**	Chinese	11
	Palestinian	1
	British	1
	French	1
	India	1
**Educational level**	Secondary or below	5
	College or above	10
**Employment status**	Employed(full-time/part-time/self-employed)	11
	Unemployed	1
	Housewife/retire	1
	Full-time students	2
**Monthly household income(HKD)**	<20,000	5
	20,000–39,999	6
	40,000–59,999	4
**Got COVID since the pandemic began**	Yes (positive COVID test)	4
	No	11
**Duty or Choice to take the COVID vaccine**	Duty	4
	Choice	9
	Prefer not to say	2

**Fig 1:**
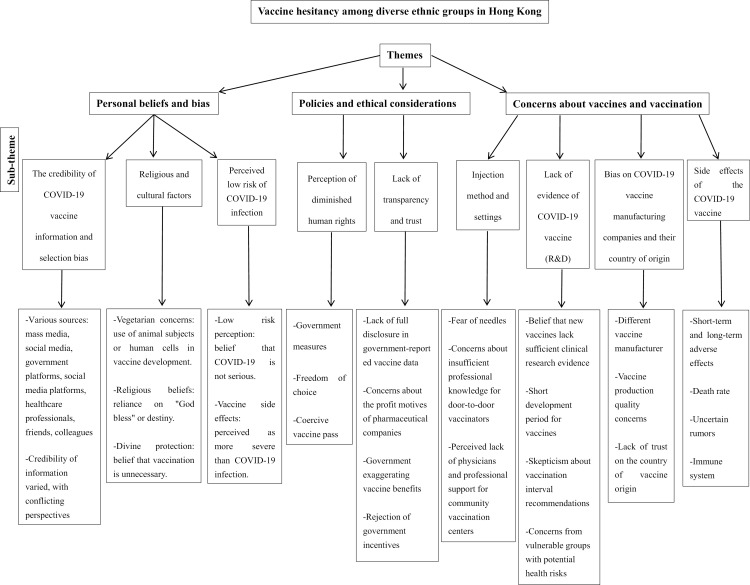
A thematic chart of the themes and sub-themes of vaccine hesitancy identified among diverse ethnic groups in Hong Kong.

Fig 1 presents the three main themes and nine sub-themes generated in this study, along with their respective exemplifications, in a thematic chart illustrating participants’ concerns and barriers toward the COVID-19 vaccine.

#### 1. Personal beliefs and biases.

##### 1.1 The credibility of COVID-19 vaccine information and selection bias.

The commonly reported sources of COVID-19 vaccine information of participants include news and articles reported on television, newspapers, government websites, and local and international social media, such as Facebook and Twitter, recommendations/suggestions provided by healthcare professionals, friends, and colleagues, as well as prestigious journal papers. They found that the information has different levels of credibility, often representing conflicting perspectives. For example, *“Information from the government told us that people will have less severe COVID-19 symptoms after receiving vaccination”* (Participant 3) but “*most of my friends and some medical staff opposed vaccination due to a lack of research to prove the efficacy and safety of vaccines*” (Participant 5 &13). While some participants regarded information from the government website as having high credibility (Participants 10 & 15), several participants were more likely to trust international information sources, because “*they report first-hand information, which is more reliable*” (Participants 8 &13). These biases they have held might impact the information they received and subsequently lead to some hesitancy toward vaccination.

##### 1.2 Religious and cultural factors.

Some participants perceived that people’s vaccination decisions were affected by religious and cultural factors. They suggested that *“some vegetarians might concern that animal subjects or human cells were integrated into the vaccine during the development of the COVID-19 vaccines, which is inconsistent with their moral code”* (Participant 9). Moreover, people with religious beliefs are more likely to believe “*God bless*” or destiny, as a male participant suggested that, *“a friend of mine thinks he does not need the COVID-19 vaccines. As he is blessed by God, he will not die if infected with the virus” [Participant 11].*

##### 1.3 Perceived low risk of COVID-19 infection.

Participants’ perceptions of a low risk of COVID-19 infection can prevent them from getting vaccinated. For example, one participant described that:

*“The corona is not that serious, that is no need to get vaccinated, it is useless. Actually, the side effects of the COVID-19 vaccine are more serious than I got infected with the COVID virus that is why I don’t think I need to take the vaccine” [Participant 1].*

#### 2. Policies and ethical considerations.

##### 2.1 Perception of diminished human rights.

Most of the participants perceived that the government’s way of restricting access to public areas and promoting vaccination for citizens are compulsory (Participant 3 & 9). Participant 3 felt that “*people’s freedom of choice was violated” and said “I think everyone should have the freedom of choice. For those who have to fulfill the vaccine pass criteria in order to work, they feel like being forced as well”.*

##### 2.2 Lack of transparency and trust.

Perceived low transparency in government-reported data about the COVID-19 vaccine and a lack of trust in the government are key factors leading to participants’ vaccine hesitancy. Some participants were worried that pharmaceutical companies might promote their vaccines to the government only for making a profit, so they felt the lab report from those companions might be unreliable: *“Sometimes the lab may change the information because they are more money-oriented”* (Participant 4). *“The government does not provide full disclosure sometimes. The government tends to exaggerate the positive side of the vaccine since they want to boost the vaccination rate. I do not believe they showed us the truth”* (Participant 3)*.* Several participants did not accept the use of incentives by the government to promote the COVID-19 vaccination in citizens. *“Physical health should not be exchanged with money, and wise people should not be attracted by incentives”* (Participant 5)*.*

#### 3. Concerns about vaccines and vaccination.

##### 3.1 Injection method and settings.

Although the majority of participants did not mention needle pain, some of them expressed their fear of pain and hoped a future vaccine would adopt the nasal spray method, for example, *“I’m a little bit scared about the needle, so I turned my head away when I was having my jab. This is a reason for me to withhold the 3rd dose”* (Participant 8)*.*

In addition, some participants had worries about providing door-to-door vaccinators and setting vaccinators in the community centre. They assumed that *“Door-to-door vaccinators might not have enough professional knowledge”* (Participant 4). They believe that healthcare staff in the hospital are more reliable and professional. It was mentioned,*“Community vaccination centre has fewer physicians to provide support. I felt uncomfortable inside the community vaccination centre, I did not have a happy mood throughout the process”* (Participant 9).

##### 3.2 Lack of evidence of COVID-19 vaccine (R&D).

A belief that new vaccines were introduced without sufficient clinical research evidence was related to vaccine hesitancy. Most participants believed that a short development period would not provide enough evidence to guide the schedule and dosage of a vaccine. *“Since the vaccine was developed in such a short time without any observation of its long-term side effects”* (Participant 6). *“The government suggested the interval between 1st and 2nd dose should be 2 weeks. I am always curious about the interval difference. Their claim is not based on scientific research or any evidence.”* (Participant 9). Some vulnerable groups, such as people with chronic illnesses and pregnant women, were worried about their health and their babies’ well-being. *“Since I have a chronic illness, I am really afraid the vaccine would bring more harm to my health”* (Participant 4). *“Some studies reported that the vaccine would lead to premature birth, so people are really scared”* (Participant 10).

##### 3.3 Bias on COVID-19 vaccine manufacturing companies and their country of origin.

Some participants preferred the German-made vaccine over the China-made one. They believed the quality standard of producing vaccines in China was low as they had heard some negative news about China products. *“Manufactures in Western countries are better, and I have less confidence in taking vaccines made in the mainland”* (Participant 4). Such lack of confidence in the China-made vaccine was another factor leading to vaccination hesitancy.

##### 3.4 Side effects of the COVID-19 vaccine.

The COVID-19 vaccine-related side effects, including body pain, particularly injection point pain, menstrual disorders, skin allergies and death rate, and unexpected long-term adverse effects, largely discouraged participants to opt-in accept the COVID-19 vaccine. As some participants discussed: *“I heard a lot of hearsay [from friends] that really bothered me. Some of my friends had rash and skin ulceration after the vaccination, but their doctors still said it is ‘okay to have the second dose. I think the vaccine might impact our immune system, although there is no evidence at this moment”* (Participant 6). *“It is an unknown for long-term side effects”* (Participant 9).

## Discussion

This study revealed that COVID-19 vaccine hesitancy among diverse ethnic groups of citizens in Hong Kong was related to personal belief and bias, policies, ethical consideration, and concerns about vaccines and vaccination. Our findings are consistent with previous publications that social media could affect people’s vaccination choices [27–29]. The recently emerging algorithm system may deliver incorrect information (fake news) and impact how social network users get data [28, 29]. Misinformation about the COVID-19 vaccination caused individuals to have misconceptions about the vaccine. For instance, the propagation of misleading news on social media led to opposition to vaccinations or an increase in vaccination apprehension [27]. Some unproven information about the potential side effects of the COVID-19 vaccine increased people’s vaccine hesitancy and fear [30]. In this study, most of the participants were unable to determine the reliability of various social media information sources, while more exposure to vaccine-critical websites may have a worse impact on vaccination intention [31]. Healthcare practitioners, as one of the most trusted sources of information identified in this study, can play a critical role in mitigating vaccine hesitancy by engaging directly with communities. Training healthcare practitioners is recommended to deliver culturally appropriate and empathetic counseling to help dispel myths and alleviate fears about vaccination among ethnic groups [28]. They can also leverage social media platforms accessible to Hong Kong’s ethnic groups (e.g., Facebook, YouTube, Twitter) to disseminate accurate information and curb the spread of misinformation [32]. Public health authorities are recommended to prioritize the visibility of official and credible sources on digital platforms to ensure that reliable information is easily accessible [33]. Interactive tools, such as hotline services or live Q&A sessions with healthcare professionals, could also address individual concerns in a safe and non-judgmental environment [34].

The religious and cultural factors were also associated with participants’ hesitancy towards regular COVID-19 vaccination in this study. The finding is consistent with Garcia and Yap [35] who found that religion combined with a proper understanding of the efficacy and efficiency of COVID-19 vaccination could positively impact people’s vaccination decisions. Our participants raised the concern from the vegetarian group that animal products/animal tissue cells might be used to create the COVID-19 vaccine, and some people believed that there is no need to get vaccines as God would guard them. However, these misunderstandings lack scientific evidence and are likely to cause vaccine hesitancy [36]. To reduce vaccine hesitancy, it is necessary to develop and implement culturally tailored interventions that address specific misconceptions and barriers faced by diverse ethnic and religious groups. For instance, public health campaigns can collaborate with religious leaders and community organizations (e.g., The Hong Kong Council of Social Service, Hong Kong Unison) to deliver scientifically accurate and transparent messages that align with cultural and religious values to foster trust and promote vaccine acceptance.

A lack of knowledge about COVID-19 and participants’ perceived low risk of infection are also linked to regular vaccine hesitancy. Our findings are aligned with a previous study showing that people were more likely to adopt other hygiene strategies, such as wearing masks, washing hands or using hand sanitizers but less likely to rely on vaccination as a key way to fighting with COVID-19, when they perceived their infection risk was low [37]. Public health strategies are recommended to focus on educating the public about the importance of vaccination as a key measure to control infectious diseases, complementing other hygiene practices like mask-wearing and handwashing. Vaccine promotion campaigns are suggested to emphasize that vaccination not only protects individuals but also facilitates the resumption of normal life by contributing to herd immunity. Specific strategies might include targeted outreach programs in schools, workplaces, and community centers to address gaps in knowledge and foster a greater sense of collective responsibility toward vaccination.

In order to rapidly control the COVID-19 pandemic, the Hong Kong government and many governments globally utilized financial incentives (e.g., coupons) [38] and compulsory social distancing strategies (e.g., a “traffic lights” system) [39] to increase the vaccination rate. However, these motivational methods raised several ethical concerns, such as diminished human rights and trust in the government. Chang et al.[40] have reported that financial incentives did not significantly increase COVID-19 vaccine uptake among vaccine-hesitant individuals. In fact, they may have negative consequences of increasing fears about the COVID-19 vaccines [40–42]. Both our study and previous studies have shown that distrust in government and public officials contributed to vaccination hesitancy [41, 42]. It is recommended that the government and vaccine manufacturers adopt a transparent and proactive approach in providing regular updates on COVID-19 vaccines, which could include clear and accessible information about vaccine effectiveness, safety, potential side effects, and complications, with a focus on addressing the specific concerns of Hong Kong’s population [43]. For example, concerns raised by local communities, such as the use of animal-derived ingredients in vaccines or fears about long-term side effects, should be directly addressed through scientific evidence and culturally respectful communication. Pharmaceutical companies could conduct additional clinical trials that include vulnerable groups specific to Hong Kong, such as children, pregnant women, and the elderly, while also considering the needs of ethnic minority groups who may face unique barriers to vaccination. More efforts are needed to make clinical trial data and vaccine information available in multiple languages, including Cantonese, English, and other languages spoken by ethnic minorities in Hong Kong (e.g., Thai, Urdu, Nepali), to build trust across communities [44].

The study has several limitations and these influence the transferability of the findings to the wider population. First, some participants were pregnant who might have different concerns about taking the COVID-19 vaccine. A majority of the interested participants were female (73%). The study only included people who could speak English and/or Cantonese. The voices of those who speak other languages and have vaccine hesitancy might be overlooked. The limitation also lies in the diversity of the participant pool, as the majority were of Chinese nationality (11 participants of Chinese nationality and 4 from ethnic minority groups). While the participants included some representation of larger ethnic minority groups in Hong Kong, resource and access constraints prevented the inclusion of more diverse communities. Future research should recruit a broader range of ethnic groups to provide a more comprehensive understanding of vaccine hesitancy. Future studies could also develop culturally-tailored interventions to address vaccine hesitancy following the identified main reasons in this study.

Another limitation that requires further research is the lack of clarity in defining and measuring vaccine hesitancy. Vaccine hesitancy has been inconsistently conceptualized, often conflating psychological indecision with vaccination behaviors, which complicates the development of targeted interventions [3]. Previous studies have described vaccine hesitancy as a psychological state of indecisiveness regarding vaccination decisions, independent of actual behavior [3, 4]. For instance, many individuals who ultimately choose to vaccinate may still experience hesitancy. Future studies may consider applying this refined view to better understand and address vaccine hesitancy (e.g., a cognitive or emotional state of indecision or behavioral indecision) in specific subpopulations, such as ethnic minorities, religious groups, and vulnerable populations in Hong Kong.

## Conclusion

Various challenges affect the successful implementation of COVID-19 vaccination programs in Hong Kong. Collaborative and concerted efforts should be contributed by the governments, health policy makers, media sources and vaccine manufacturers and various stakeholders to decrease people’s hesitancy towards regular COVID-19 vaccinations. The Hong Kong government is recommended to build COVID-19 vaccination trust among the general public, via the spread of timely and clear messages through trusted channels advocating the safety and efficacy of currently available COVID-19 vaccines. The study findings have important implications for public health interventions to tackle vaccine hesitancy in Hong Kong and other ethnically diverse regions in the world.
